# Intramedullary reaming and irrigation and antibiotic-loaded calcium sulfate implantation for the treatment of infection after intramedullary nailing: a retrospective study of 19 cases

**DOI:** 10.1186/s12891-020-03734-z

**Published:** 2020-10-28

**Authors:** Hong-An Zhang, Chun-Hao Zhou, Xiang-Qing Meng, Jia Fang, Cheng-He Qin

**Affiliations:** 1 Department of Orthopaedics and Traumatology, Guangdong second provincial general hospital, The Second Clinical Medical School of Southern Medical University, Guangzhou, 510317 P.R. China; 2 Department of Orthopaedics, Nanfang Hospital, Division of Orthopaedics and traumatology, Southern Medical University, Guangzhou, 510515 P.R. China

**Keywords:** Debridement, Fracture-related infection, Intramedullary nailing, Local antibiotic delivery, Reaming

## Abstract

**Background:**

The incidence of intramedullary infection is increasing with increased use of intramedullary fixation for long bone fractures. However, appropriate treatment for infection after intramedullary nailing is unclear. The purpose of this study was to report the results of our treatment protocol for infection after intramedullary nailing: intramedullary nail removal, local debridement, reaming and irrigation, and antibiotic-loaded calcium sulfate implantation with or without segmental bone resection and distraction osteogenesis.

**Methods:**

We retrospectively reviewed the records of patients with an infection after intramedullary nailing treated from 2014 to 2017 at our center. Patients with follow-up of less than 24 months, received other treatment methods, or those with serious medical conditions were excluded from the analysis. Patients met the criteria were treated as described above, followed by distraction osteogenesis in 9 cases to repair bone defect. The infection remission rate, infection recurrence rate, and post-operative complication rates were assessed.

**Results:**

A total of 19 patients were included in the analysis. All of patients had satisfactory outcomes with an average follow-up of 38.1 ± 9.4 months (range, 24 to 55 months). Eighteen patients (94.7%) achieved infection remission; 1 patient (5.3%) developed a reinfection that resolved after repeat debridement. Nine patients with bone defects (average size 4.7 ± 1.3 cm; range, 3.3 to 7.6 cm) were treated with bone transport which successfully restored the length of involved limb. The mean bone transport duration was 10.7 ± 4.0 months (range, 6.7 to 19.5 months). The majority of patients achieved full weight bearing and became pain free during the follow-up period. Postoperative complications mainly included prolonged aseptic drainage (7/19; 36.8%), re-fracture (1/19; 5.3%) and joint stiffness, which were successfully managed by regular dressing changes and re-fixation, respectively.

**Conclusion:**

Intramedullary nail removal, canal reaming and irrigation, and antibiotic-loaded calcium sulfate implantation (with or without distraction osteogenesis) is effective for treating infections after intramedullary nailing.

## Introduction

Infection after intramedullary nailing is uncommon, with a reported rate of 0.9 to 3.8% [1, 2]. In a retrospective analysis of more than 1000 cases of tibial shaft fractures treated with intramedullary nailing, the infection rate after treatment of closed fractures was 1.9%, and the infection rate after treatment of open fractures was 7.7% [3, 4]. Although the infection rate is not high, if an infection is not treated in a timely manner, complications including osteomyelitis, fracture non-union, physical disability, or even systemic sepsis are inevitable.

The management of this type of infection remains controversial [5, 6]. Makridis et al. [7] described 3 stages of infection after intramedullary nailing: Stage I; 2–6 weeks after operation manifesting as cellulitis, Stage II; 2–9 months after operation, manifesting as delayed wound healing, exudation, osteonecrosis, and pathological fracture, Stage III; 9 months or longer after operation manifesting as definite osteomyelitis. Each stage has its own management protocols. For the treatment of Stage I infections, a more conservative approach is widely accepted [5, 7]_._ However, opinions on the best treatments for Stage II and III infections vary greatly [1, 7, 8], especially when the fracture hasn’t healed yet. Unfortunately, to date there are no uniform and standard treatment protocol for Stage II and III intramedullary infections [5, 9]. Thus, the management of infections after intramedullary nailing tend to be experience-based, rather than evidence-based protocols.

In our experience, an intramedullary nail as a foreign fixator lacks an adequate blood supply, which means systemic antibiotics cannot reach the interface of the nail to eradicate the biofilm bacteria effectively [10]. Furthermore, delayed or inappropriate treatments allow the pathogens to spread through the whole medullary canal resulting in a diffuse infection [10]. Therefore, we believe that nail removal and surgical debridement are vital for the treatment of Stage II and III infections after intramedullary nailing, as it destroys the biofilm produced by bacteria, and thus enhances the efficiency of antibiotics which in turn improves the infection remission rate. Surgical debridement mainly includes local debridement and intramedullary nail management. Intramedullary reaming and irrigation are important components of surgical debridement, since the process eliminates the endosteal sequestra of the canal, lowers the intraosseous pressure, improves vascularization of the bone, and removes the bacterial biofilm [11]. The effectiveness of reaming and irrigation for the treatment of intramedullary nail infections and osteomyelitis has been reported in prior studies [12–14].

After debridement, there may be remaining bacterial residue in the marrow canal or the surrounding soft tissue, and it only takes about 72 h for a bacterial biofilm to develop and become mature [15]. Due to the poor blood supply and osteonecrosis in osteomyelitis, administration of systemic antibiotics is usually not enough to eradicate the infection, and sometimes have little effect [16]. Antibiotic-impregnated calcium sulfate is an absorbable local antibiotic delivery system, and exhibits excellent osteogenesis and drug-loading properties. The advantages of antibiotic-loaded materials include more accurate positioning, higher local antibiotic concentration, less side effects, and longer treatment duration [17]. Studies have shown good outcomes when antibiotic-loaded calcium sulfate is used for the treatment of bone infections [18, 19].

Based on literature data and our experience, we have developed a protocol for the treatment of infections after intramedullary nailing: intramedullary nail removal, local debridement, medullary canal reaming and irrigation, and antibiotic-loaded calcium sulfate implantation, with or without secondary osteotomy and distraction osteogenesis. The purpose of this study is to describe this technique and report our results using the protocol for treating infections after intramedullary nailing.

## Materials and methods

The records of patients diagnosed with an infection after intramedullary nail fixation and treated at our center from 2014 to 2017 were retrospectively analyzed. The inclusion criteria for study were: 1) Infection after initial intramedullary nail fixation and the intramedullary nail was in place when admitted to the hospital; 2) Treated as per our protocol described above; 3) Minimum follow-up of 24 months. The exclusion criteria were: 1) Patients with severe liver or kidney dysfunction, cardiovascular disease, or diabetes with uncontrolled blood glucose; 2) Patient was not treated with our described method; 3) Follow-up < 24 months. Diagnosis of infection after intramedullary nailing was based on the clinical criteria described by Metsemakers et al. [20], which include the presentation of 1) a fistula, sinus, or wound breakdown, or 2) purulent drainage from the wound. Of course, we also combined the presence of positive biochemical infection markers, imaging results, and culture and histology results of tissue samples collected during surgery.

Before surgical interventions, patients were informed with the details of treatment protocols, and the informed consents were signed by patients themselves. Our study was approved by the Ethical Committee of Guangdong Second Provincial General Hospital and Nanfang Hospital.

### Surgical technique

The intramedullary nail was removed first, and subsequent debridement procedures were based on whether or not the fracture had healed.

In the case of healed fractures, the entry point of the intramedullary nail was enlarged. Since the reamer-irrigator-aspirator (RIA) system is not available in our country, irrigation fluid cannot be aspirated directly. Therefore, distal diaphysis fenestration was performed to allow drainage of the irrigation fluid and necrotic tissues. The medullary canal was reamed repeatedly with a larger-diameter reamer head (size based on preoperative measurements and information of the initial surgery) to completely debride necrotic tissue. Local debridement was also performed which consisted of removal of all infected bone, soft tissue, and any sinuses. Segmental bone resection was not performed in cases with healed fractures, since none of cases presented with diffuse osteomyelitis according to the Cierny-Mader classification [21].

For cases without bone union, after intramedullary nail removal the fracture site was segmentally resected to reduce the possibility of infection recurrence [22]. Bony defects in these cases were managed by osteotomy and distraction osteogenesis in a secondary surgery, after markers of inflammation had returned to normal levels.

Surrounding soft tissues were also debrided. In all patients, samples were sent for culture and pathological examination. After radical debridement, the medullary canal was irrigated with saline using an impulsive irrigation gun. Elimination of the dead space caused by removal of the intramedullary nail and reaming was directly filled with antibiotic-loaded calcium sulfate (Stimulan, Biocomposites Ltd.). The antibiotic-loaded calcium sulfate was prepared with a ratio of vancomycin 0.5 g + gentamycin 2 ml + calcium sulfate 5 cm^3^. An appropriate amount of saline was added to the mixture, and the calcium sulfate preparation was injected into the medullary canal and the segmental bone defect. Considering the economic situations of patients and the potential cytotoxic effect of calcium sulfate, the total volume of the calcium sulfate preparation used per patient was no more than 50 ml. Vancomycin and gentamicin were both used in the preparation, in order to cover both Gram-positive and -negative bacteria.

After implantation, an external fixator was used in 13 cases for avoiding debridement-related fracture (4 cases) or secondary distraction osteogenesis in 9 cases with bone defects.

### Postoperative management

Intravenous antibiotics were empirically administrated until culture results were available, and then adjusted based on the results. Due to the application of antibiotic-loaded calcium sulfate, the total course of systemic antibiotics was less than 2 weeks. Patients with bony defects received an osteotomy in a secondary surgery, and bone transport was carried out 1 week after the surgery, with an initial rate of 1 mm/day, and then regulated according to the rate of bone formation and patient feedback. During follow-up, patients received testing of white blood cell (WBC) count, C-reactive protein (CRP) and erythrocyte sedimentation rate (ESR), and standard anteroposterior (AP) and lateral radiographs to determine the effectiveness of infection elimination and bone formation.

External fixators were removed once bones were determined to be strong enough for weight bearing. Walking-aids were recommended for the first several weeks after the operation and 1 month after external fixation removal for patients who received segmental bone resection and bone transport. For patients without bone transport, however, early rehabilitation training without any assistance was encouraged.

### Outcome evaluation

Infection Remission was defined as the absence of any positive markers of infection, no evidence of infection on radiographs or physical examination, and a completely healed wound. Bone Union was assessed by the formation of new bone on radiographs. Infection Recurrence was defined by the presence of positive clinical symptoms, radiographic findings, and elevated levels of inflammatory markers. Continuous drainage without signs of a local infection for more than 1 month was defined as prolonged aseptic drainage.

## Results

A total of 19 patients who met the inclusion criteria were included in the analysis. There were 15 men and 4 women with a mean age of 39.0 ± 10.1 years (range, 23 to 56 years). The femur was involved in 7 patients and tibia in 12 patients, and all fractures were due to some form of trauma (traffic trauma in10 cases, falling injury in 6 cases and heavy pound injury in 3 cases). Nine patients suffered from infection on right extremity while 10 patients on left extremity. All patients presented with a sinus at their first physical examination, and preoperative radiographs indicated that 10 patients had healed fractures and 9 patients had unhealed fractures. The mean preoperative ESR was 49.4 ± 34.2 mm/h, mean CRP level was 33.1 ± 23.8 mg/L, and the mean WBC count was 12.75 ± 6.63 × 10^9^/L. Patient preoperative data are summarized in Table 1. In all cases, no antibiotics were administered until after specimens were obtained for culture during surgery.

**Table 1:** Preoperative characteristics of nineteen cases

Case No.	Age	Initial trauma	Site/Side	Open or closed fracture	history (months)	Sinus	Fracture healed or not	Infection markers before surgery
1	44	Falling height	Femur/R	Closed	36	Yes	Yes	WBC: 6.4 × 10^9^ CRP: 56.7 ESR: 14
2	52	Traffic trauma	Tibia/L	Open	13	Yes	Yes	WBC: 5.7 × 10^9^ CRP: 9 ESR: 26
3	33	Falling height	Tibia/R	Closed	3	Yes	No	WBC: 11.3 × 10^9^ CRP: 77 ESR: 101
4	37	Traffic trauma	Femur/R	Open	48	Yes	Yes	WBC: 6.8 × 10^9^ CRP: 9.4 ESR: 37
5	24	Falling height	Tibia/R	Open	6	Yes	Yes	WBC: 6.3 × 10^9^ CRP: 5.3 ESR: 29
6	26	Falling height	Tibia/L	Closed	14	Yes	No	WBC: 5.7 × 10^9^ CRP: 7.4 ESR: 45
7	44	Heavy pound injury	Tibia/L	Open	4	Yes	No	WBC: 10.5 × 10^9^ CRP: 23.2 ESR: 15
8	44	Traffic trauma	Femur/L	Closed	20	Yes	Yes	WBC: 18.3 × 10^9^ CRP: 37.3 ESR: 114
9	47	Traffic trauma	Femur/R	Open	36	Yes	Yes	WBC: 12.6 × 10^9^ CRP: 35.3 ESR: 89
10	45	Falling height	Tibia/L	Open	40	Yes	Yes	WBC: 10.3 × 10^9^ CRP: 42.6 ESR: 67
11	52	Heavy pound injury	Tibia/L	Closed	5	Yes	Yes	WBC: 7.5 × 10^9^ CRP: 18.3 ESR: 5
12	37	Traffic trauma	Tibia/L	Open	19	Yes	No	WBC: 22.1 × 10^9^ CRP: 51.0 ESR: 78
13	46	Traffic trauma	Femur/L	Open	13	Yes	No	WBC: 9.3 × 10^9^ CRP: 11.3 ESR: 25.7
14	27	Traffic trauma	Femur/R	Closed	15	Yes	Yes	WBC: 17.7 × 10^9^ CRP: 77.5 ESR: 32
15	34	Traffic trauma	Tibia/R	Open	30	Yes	Yes	WBC: 28.3 × 10^9^ CRP: 44.9 ESR: 29
16	56	Falling height	Tibia/L	Closed	7	Yes	No	WBC: 8.1 × 10^9^ CRP: 65.3 ESR: 29
17	23	Traffic trauma	Tibia/R	Open	11	Yes	Yes	WBC: 17.5 × 10^9^ CRP: 10.6 ESR: 76
18	28	Traffic trauma	Femur/R	Closed	11	Yes	Yes	WBC: 21.8 × 10^9^ CRP: 28.9 ESR: 23
19	42	Falling height	Tibia/L	Closed	17	Yes	No	WBC: 16.1 × 10^9^ CRP: 17.3 ESR: 103

Thirteen patients required external fixation, and 9 of these patients received segmental bone resection and bone transport after the infection markers became normal, and started bone transport 1 week later (Fig. 3) aiming to repair the bone defect. The average bone defect was 4.7 ± 1.3 cm (range, 3.3 to 7.6 cm). Four patients who did not receive segmental bone resection were treated with external fixation to assist weight bearing because bone debridement was very extensive. Postoperatively, clindamycin, cephalosporins, or quinolones were the most commonly administered intravenous antibiotics, with a mean duration of 8.3 ± 3.2 days (range, 3–14 days). Conventionally, a total course of antibiotics includes 2 weeks of for intravenous administration, and an additional route and another 4 weeks of oral administration. In our protocol, we omitted the 4 weeks of oral antibiotic administration because we deemed the extremely high concentrations and fairly long curative duration produced by the local antibiotic delivery system was enough to eradicate residual bacteria. The mean time for normalization of infection markers after surgery was 3.4 ± 1.7 weeks (range, 2 to 8 weeks).

**Fig. 1:**
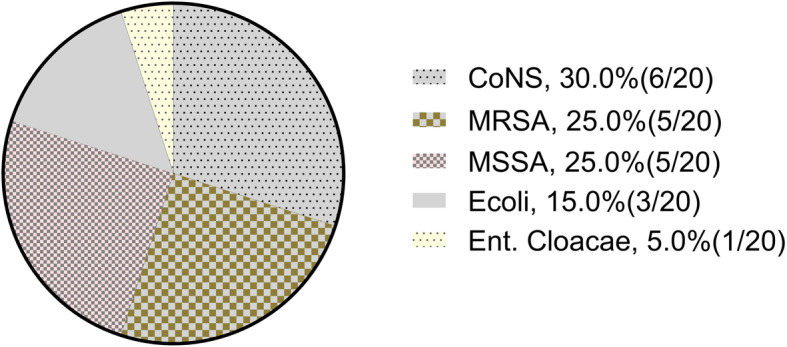
Distribution of bacterial culture results after operation

A total of 20 strains of bacteria were isolated from 17 cases, with a positive culture rate of 89.5% (17/19). Coagulase-negative *Staphylococcus* (30.0%, 6/20) was the most commonly isolated pathogen, followed by methicillin-resistant *Staphylococcus aureus* (MRSA) (25.0%, 5/20), methicillin-sensitive *Staphylococcus aureus* (MSSA) (25.0%, 5/20), and *Escherichia coli* (15%, 3/20). Polymicrobial infections were identified in 3 patients (15.8%, 3/19). The distribution of bacterial culture results are shown in Fig. 1.

**Fig. 2:**
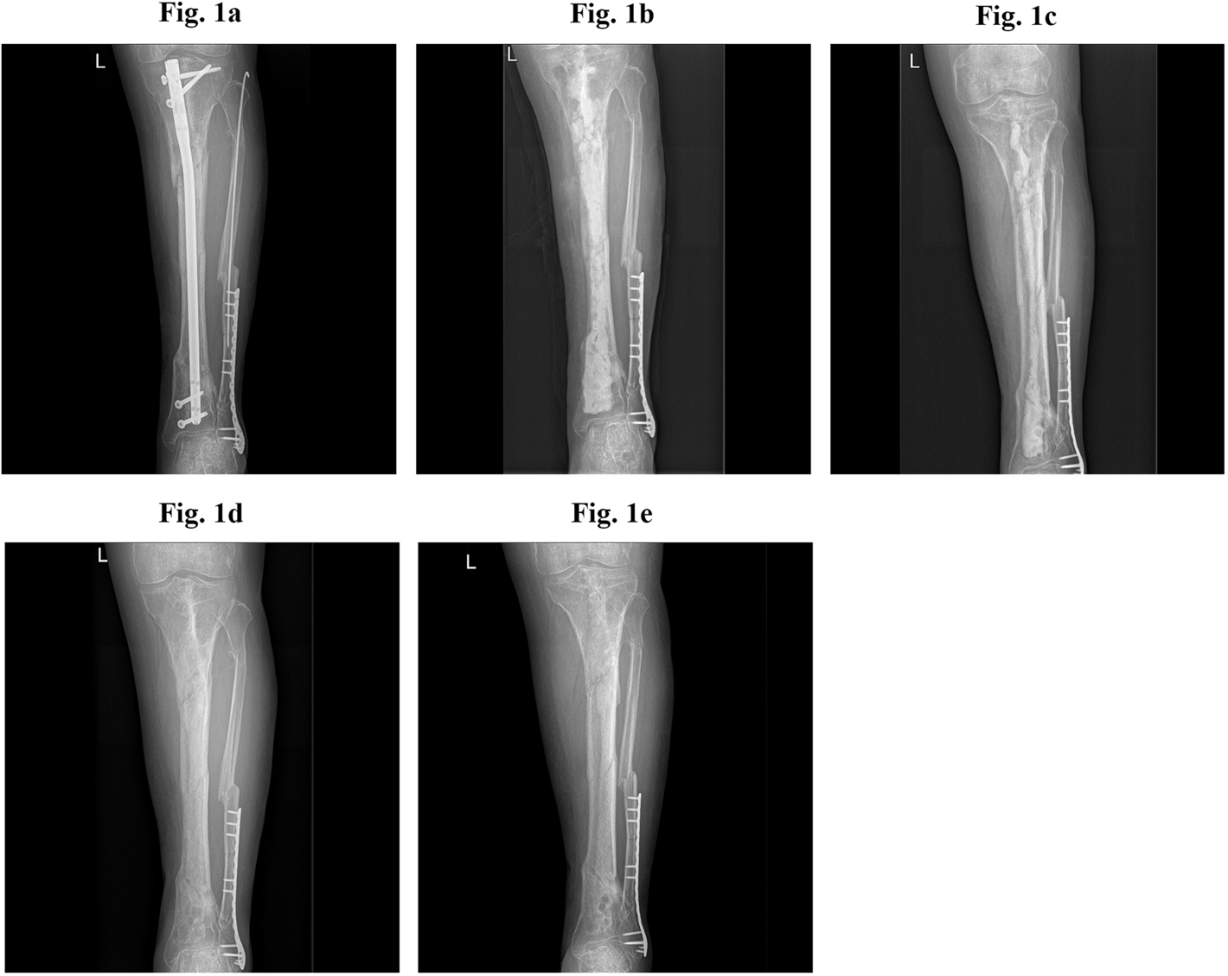
Representative case of intramedullary nail removal, debridement, and application of antibiotic-loaded calcium sulfate

**Fig. 3:**
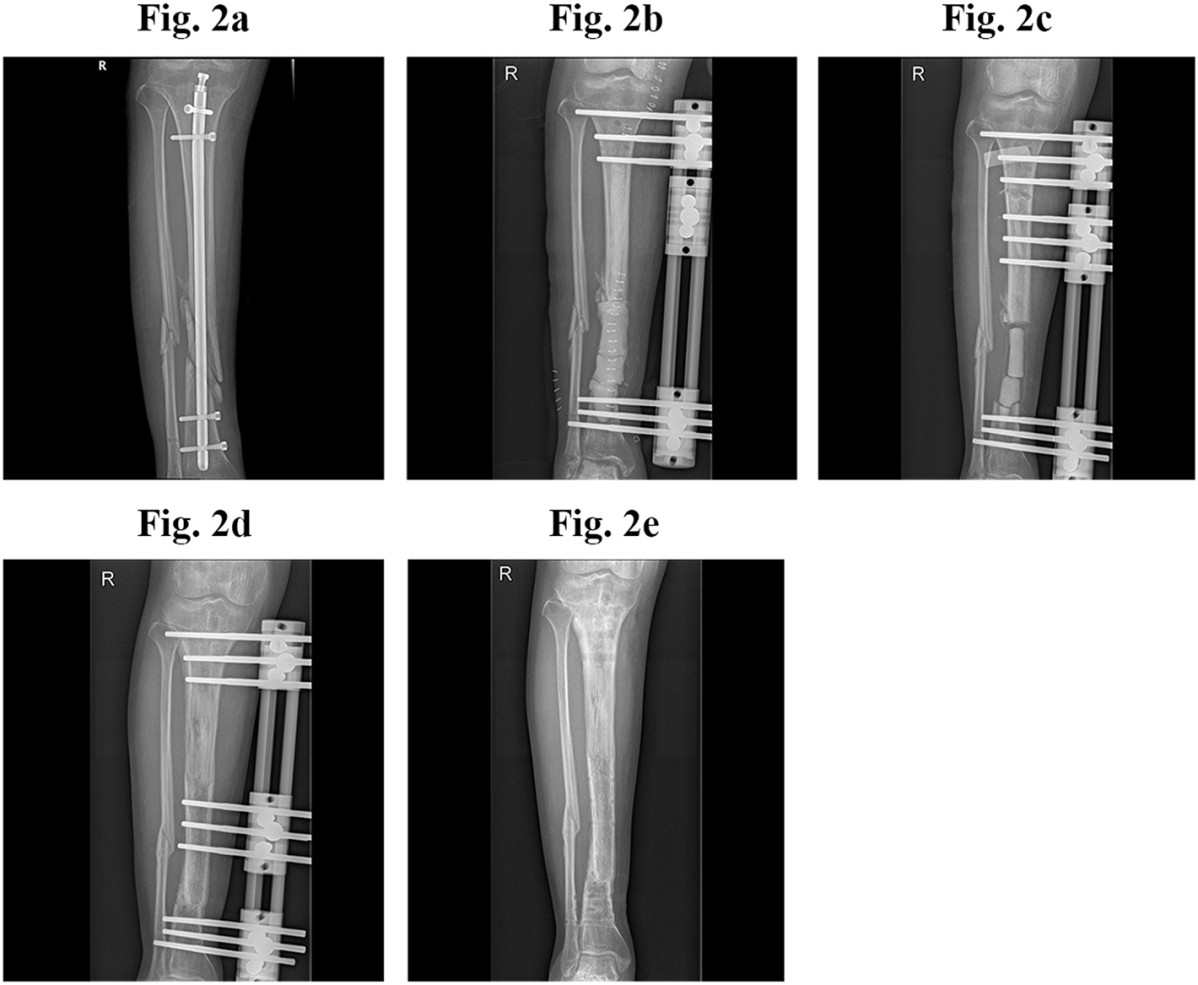
Representative case of intramedullary nail removal, debridement, application of antibiotic-loaded calcium sulfate, external fixation, and bone transport

With a mean follow-up of 38.1 ± 9.4 months (range, 24 to 55 months), 18 (94.7%) patients achieved infection remission after the first surgical treatment, while 1 patient (5.3%) developed infection recurrence 3 months after surgery and underwent segmental resection and bone transport. The representative cases were presented in Figs. 2 and 3. Twelve (63.2%) patients became completely pain-free, and 16 (84.2%) patients achieved full weight bearing during the follow-up period. One patient (5.3%) experienced a re-fracture 4 months after surgery, and was successfully treated with external fixation. Prolonged aseptic drainage was the most frequent postoperative complication, and occurred in 7 patients (36.8%). In all cases, the drainage was successfully treated with regular local wound care and dressing changes. Bone transport was successful in all patients, and the mean fixation duration was 10.7 ± 4.0 months (range, 6.7 to 19.5 months). attributing to early function rehabilitation, only 1 (5.3%) case was recorded with joint stiffness after bone transport. Surgery and follow-up data are summarized in Table 2.

**Table 2:** The details of surgery and follow-up outcomes of nineteen cases

Case No.	Microbiology	Description of surgery	Time for normalization of infection markers	Follow-up (months)	Recurrence	Outcome
1	MRSA	IM nail removal+Debridement+CS + EF	2 weeks	36	No	Pain free, FWB mobilisation
2	*E. coli*	IM nail removal+Debridement+CS	4 weeks	24	No	Pain free, FWB mobilisation
3	CoNS +E. coli	IM nail removal+Debridement+CS + EF+ Bone transport	8 weeks	38	No	Mild pain with movement, FWB mobilisation
4	–	IM nail removal+Debridement+CS	4 weeks	37	No	Pain free, FWB mobilisation
5	MSSA	IM nail removal+Debridement+CS + EF+ Bone transport	3 weeks	27	No	Mild pain and movement limitation on right ankle
6	MRSA	IM nail removal+Debridement+CS + EF+ Bone transport	2 weeks	26	No	Pain free, FWB mobilisation
7	CoNS	IM nail removal+Debridement+CS + EF	5 weeks	41	Yes, 3 months after the first surgery	Segmental resection and bone transport
8	Ent. Cloacae	IM nail removal+Debridement+CS	2 weeks	46	No	Pain free, FWB mobilisation
9	–	IM nail removal+Debridement+CS	4 weeks	25	No	Refracture four months after first surgery, followed by EF fixation
10	CoNS	IM nail removal+Debridement+CS + EF+ Bone transport	2 weeks	33	No	Pain free, FWB mobilisation
11	MSSA	IM nail removal+Debridement+CS + EF	3 weeks	52	No	Pain free, FWB mobilisation
12	CoNS	IM nail removal+Debridement+CS + EF+ Bone transport	3 weeks	41	No	Mild pain with movement, FWB mobilisation
13	MSSA	IM nail removal+Debridement+CS	2 weeks	38	No	Pain free, FWB mobilisation
14	MSSA	IM nail removal+Debridement+CS + EF	2 weeks	40	No	Pain free, FWB mobilisation
15	MSSA	IM nail removal+Debridement+CS + EF+ Bone transport	3 weeks	30	No	Mild pain with movement, FWB mobilisation
16	MRSA	IM nail removal+Debridement+CS + EF	2 weeks	55	No	Pain free, FWB mobilisation
17	E.coli+MRSA	IM nail removal+Debridement+CS	2 weeks	44	No	Pain free, FWB mobilisation
18	CoNS	IM nail removal+Debridement+CS + EF+ Bone transport	6 weeks	37	No	Mild pain with movement, FWB mobilisation
19	CoNS+MRSA	IM nail removal+Debridement+CS + EF+ Bone transport	6 weeks	54	No	Pain free, FWB mobilisation

*CoNS* coagulase-negative staphylococcus, *E. coli Escherichia coli*, *Ent. Cloacae Enterobacter cloacae*, *MRSA* methicillin-resistant *Staphylococcus aureus*, *MSSA* methicillinsensitive *Staphylococcus aureus*

CS, calcium sulfate; EF, external fixator; IM, intramedullary

CRP, C-reactive protein; ESR, erythrocyte sedimentation rate; WBC, white blood cells FWB, full weight bearing

## Discussion

Infection after intramedullary nailing is defined as a confined or diffuse infection of medullary cavity caused by the invasion of pathogens during intramedullary nailing. Although uncommon, it often leads to serious consequences if not treated timely and appropriately. Today’s treatment protocols of such disease vary widely according to the severity of infection and doctor’s experience, and a wide-accepted method remains unclear. In our study, we hold a more aggressive treatment method on the management of intramedullary infection since we put the infection controlling on a higher priority.

After surgical debridement and antibiotic-loaded calcium sulfate implantation, 94.7% of patients achieved infection remission after surgical treatment, while only 1 patient developed a recurrence of infection and required a second surgery. We speculate that the high remission rate is attributed to radical debridement and the use of a local antibiotic delivery system. This method thoroughly removes infected tissues and eliminates residual bacteria due to a high local antibiotic concentration and long treatment duration. Such remarkable result was similar to the previous reports. Kanakaris et al. [23] performed intramedullary nail removal, intramedullary debridement with a RIA device, and placement of antibiotic-loaded cement rods for the treatment of 24 patients with infections. The cement rods were removed once the infections were controlled, and with mean follow-up of 21 months 23 (96%) patients had no evidence of recurrent infection. The difference between their study and ours lies in the use of a RIA device and antibiotic cement. The RIA system is a device that was initially developed to prevent fat embolism and lessen the systemic inflammatory process after reaming the femur in nailing procedures [24, 25]. Due to its versatility, it has been expanded to the treatment of long bone osteomyelitis [26, 27]. Unfortunately, the RIA system is not available in our country; however, the method we used produces similar results as the use of a RIA system. The other difference between the studies is the topical antibiotic carrier. Although antibiotic-loaded cement used in medullary infection have been well-demonstrated in former studies [7, 23, 27], one of the shortcomings of such material was non-absorbable after implantation, thus required a second procedure to remove it because leaving the rods in place can increase the risk of recurrent infection. Additionally, the antibiotic level curve produced by such antibiotic cement rod has been shown to be unstable, which might lead to a sharp decrease of antibiotic level several days after implantation, resulting in incomplete eradication of the pathogens, or even becoming a nidus for bacterial colonization. In another case series, Qiang et al. [28] treated infections after intramedullary nailing in 19 patients with nail removal, reaming and irrigation, and antibiotic-loaded cement implantation. All 19 patients achieved remission from infection, although there was 1 case of non-union and 1 patient ultimately required amputation due to severe trauma. However, The shortcoming in his study was similar. As the PMMA cement was absorbable, a second surgery was necessary for cement removal.

Nine of our 19 patients were treated with additional segmental bone resection and bone transport, and all of patients achieved infection remission, and the lengths of the involved limbs were well-restored. Although our outcomes were satisfactory, the best protocol for the management of non-healing fractures is not clear and treatments are primarily based on the experiences of individual surgeons. Those who prefer to retain the nails believe that fracture healing is more important, and that management of intramedullary infection can be postponed until after bone union. On the other hand, some surgeons believe infection control should take priority. Thus, based on different concepts treatment protocols mainly include 1) local debridement and antibiotic administration and retaining the nail until after bone union, 2) nail removal, re-reaming, and replacement of a larger diameter intramedullary nail or a resorbable antibiotic coated nail [10, 23], and 3) nail removal, segmental bone resection, reaming and irrigation, and bone defect reconstruction with bone transport [29] or the Masquelet technique [30, 31]. We are inclined to a more aggressive treatment protocol, and in our opinion retaining the nail or replacing it with another internal fixation device after debridement is not suitable for infection control, because the residual pathogens and its biofilms might lead a higher potential for infection recurrence and treatment failure [6]. External fixation avoids this shortcoming and remains the recommendation for treatment of bone infections [6, 11]. Additionally, a persistent infection or infection recurrence might prevent the bone healing process [32], and even lead to diffuse osteomyelitis and resulting disability and amputation. External fixation combined with segmental bone resection and transport as a mature and efficient technique can be used to manage infection, nonunion and bone defects, and deformity at the same time.

Local antibiotic-carriers were proposed in 1970s [33, 34], and are currently recommended as a bone substitute in the management of bone defects, or as a local antibiotic carrier in the case of bone infection. In our study, we used an injectable antibiotic-loaded calcium sulfate, which overcomes the shortcomings of PMMA cement, such as difficulty in intramedullary placement, the need for a second surgery for removal, and an unstable antibiotic release curve. As an absorbable antibiotic carrier, calcium sulfate has a stable antibiotic release curve, and can maintain the local antibiotic level higher than the MIC for 6–8 weeks. The local concentration is 100 to 1000 times higher than the antibiotic levels resulting from intravenous administration [35], which is sufficiently high to penetrate the bacterial biofilm. Furthermore, calcium sulfate exhibits a similar microstructure to cancellous bone and after being absorbed a network structure remains and trabecular bone can be observed under a light microscope, which contributes to the growth and migration of blood vessels and bone cells [36, 37]. To the best of our knowledge, there are no other studies reporting the use of antibiotic-loaded calcium sulfate to treat the medullary canal after removal of an intramedullary nail because of an infection.

Prolonged aseptic drainage was the most frequent complication in our study, with a relatively high rate of 36.8%, with prior studies reporting rates ranging from 4.2 to 33% [38–40]. In our experience, poor soft tissue coverage, scar formation, and excessive calcium sulfate implantation may be the reasons for the high incidence of postoperative exudation. Although this type of aseptic exudation is not a sign of infection, management is important as a persistently wet gauze can increase the risk of a wound infection. Generally, routine treatment of prolonged drainage includes regular dressing and wound care. Other effective methods to prevent prolonged aseptic drainage may include good soft tissue coverage and reduction of the amount of calcium sulfate implanted. Other complications of the treatment of intramedullary nail infection include hypercalcemia, debridement-related fracture, and post-operative pain and joint stiffness; however, these complications were rare in our series.

There are limitations of this study that need to be considered. Firstly, our outcomes were not compared with those of other surgical methods. In addition, some detailed patient data were not available because of the retrospective nature of the study, and this might influence the understanding of outcomes. Finally, our patients were heterogeneous, with different sites of infection (tibia or femur), infection with bone union or non-union, and receiving bone transport or not, all of which inevitably can lead to more complex outcomes. However, we have to point out that the emphasis of our study was to introduce an effective method to eliminate infection after intramedullary nailing, and from this perspective all patients received the same management and overall the outcomes were good.

## Conclusion

Intramedullary nail removal, medullary reaming and irrigation, and antibiotic-loaded calcium sulfate implantation seems effective in the treatment of infection after intramedullary nailing. Additional prospective studies with larger case numbers are necessary to confirm our findings.

## Data Availability

The data used and analyzed during the current study is available from the corresponding author on reasonable request.

## Abbreviations

CoNS: coagulase-negative staphylococcus
*E. coli*: *Escherichia coli*
Ent. Cloacae: *Enterobacter cloacae*
MRSA: methicillin-resistant *Staphylococcus aureus*
MSSA: methicillin sensitive *Staphylococcus aureus*
CS: calcium sulfate
EF: external fixator
IM: intramedullary
CRP: C-reactive protein
ESR: erythrocyte sedimentation rate
WBC: white blood cells
FWB: full weight bearing
